# The V protein in oncolytic Newcastle disease virus promotes HepG2 hepatoma cell proliferation at the single-cell level

**DOI:** 10.1186/s12885-023-10815-4

**Published:** 2023-04-17

**Authors:** Zhili Chu, Sihui Yang, Qianru Li, Jianing Shang, Zilong Ren, Feng Ren

**Affiliations:** 1 Xinxiang Key Laboratory of Pathogenic Biology, Department of Pathogenic Biology, School of Basic Medical Sciences, Xinxiang Medical University, Xinxiang, China; 2 Henan International Joint Laboratory of Immunity and Targeted Therapy for liver-intestinal Tumors, Xinxiang Medical University, Xinxiang, China

**Keywords:** Newcastle disease virus, V protein, Cell proliferation, MAPK signaling pathway, WNT signaling pathway

## Abstract

**Background:**

Newcastle disease virus (NDV) is an oncolytic virus that can inhibit cancer cell proliferation and kill cancer cells. The NDV nonstructural V protein can regulate viral replication; however, whether the V protein contributes to NDV oncolysis is unclear.

**Results:**

This study revealed that NDV inhibited tumor cell proliferation and that V protein expression promoted the proliferation of HepG2 cells, as determined at the single-cell level. In addition, to identify the regulatory mechanism of the V protein in HepG2 cells, transcriptome sequencing was performed and indicated that the expression/activation of multiple cell proliferation-related genes/signaling pathways were changed in cells overexpressing the V protein. Hence, the MAPK and WNT signaling pathways were selected for verification, and after blocking these two signaling pathways with inhibitors, the V protein promotion of cell proliferation was found to be attenuated.

**Conclusions:**

The results showed that the V protein regulated the proliferation of cancer cells through multiple signaling pathways, providing valuable references for future studies on the mechanism by which the V protein regulates cancer cell proliferation.

**Supplementary Information:**

The online version contains supplementary material available at 10.1186/s12885-023-10815-4.

## Introduction

Newcastle disease virus (NDV) is an oncolytic virus in the Paramyxoviridae family. NDV replicates in human tumor cells but largely fails to replicate in noncancerous human cells [1], which causes the virus to selectively kill cancer cells. NDV is an enveloped virus with a negative-strand RNA genome that encodes six structural viral proteins: nucleocapsid protein (NP), phosphoprotein (P), matrix protein (M), fusion protein (F), hemagglutinin-neuraminidase (HN), and large polymerase protein (L). These genes are arranged in the genome in the following order: 3′-NP-P-M-F-HN-L-5′. Moreover, NDV encodes two nonstructural proteins, V and W, which are produced by RNA editing during *P* gene transcription [2].

Among the viral structural proteins, NP encapsulates genomic RNA and binds to the P and L proteins to form ribonucleoprotein complexes (RNPs), which are templates for viral genome transcription and replication [3]. HN is critical for activating the F protein and forming a complex that mediates membrane fusion [4], and the M protein is required for fusion and release of nucleocapsid into the host cytoplasm [5]. In addition, NDV nonstructural proteins, particularly the V protein, play important roles in viral replication. V protein can target the IFN signaling pathway; however, type I IFN signaling is impaired, and whether the V protein can regulate viral replication in tumor cells remains to be determined.

During oncolysis, NDVs are toxic to human tumor cell lines of ecto-, endo-, and mesodermal origin, and the cytotoxicity induced by these NDV strains in tumor cells is related to multiple caspase-dependent pathways of apoptosis, which are activated independent of IFN signaling [6]. In general, apoptosis of host cells can inhibit viral replication, and the V protein is known for its antiapoptotic activity [7–9]. In our previous study, we showed that the V protein can activate MEK/ERK signaling in HeLa cells [10]. Another study showed that the V protein can upregulate cytokine signaling 3 expression, and cytokine signaling 3 overexpression in chicken embryo fibroblast cells can activate the MEK/ERK signaling pathway [11]. ERK is critical for the activation of several downstream molecules, many of which directly affect cell proliferation, survival and differentiation [12], and an increasing number of studies have suggested that MEK/ERK is a therapeutic target in human cancer [13]. Whether the V protein can regulate cancer cell proliferation is still unknown.

The present study evaluated the possible mechanisms underlying the antiapoptotic effects of the V protein, and the results suggested that the V protein may be involved in regulating cancer cell proliferation. Moreover, the role played by the V protein in regulating cancer cell proliferation was confirmed, and the results indicated that the V protein can activate multiple signaling pathways to promote cancer cell replication.

## Results

### NDV inhibits HepG2 cell proliferation

**Fig. 1:**
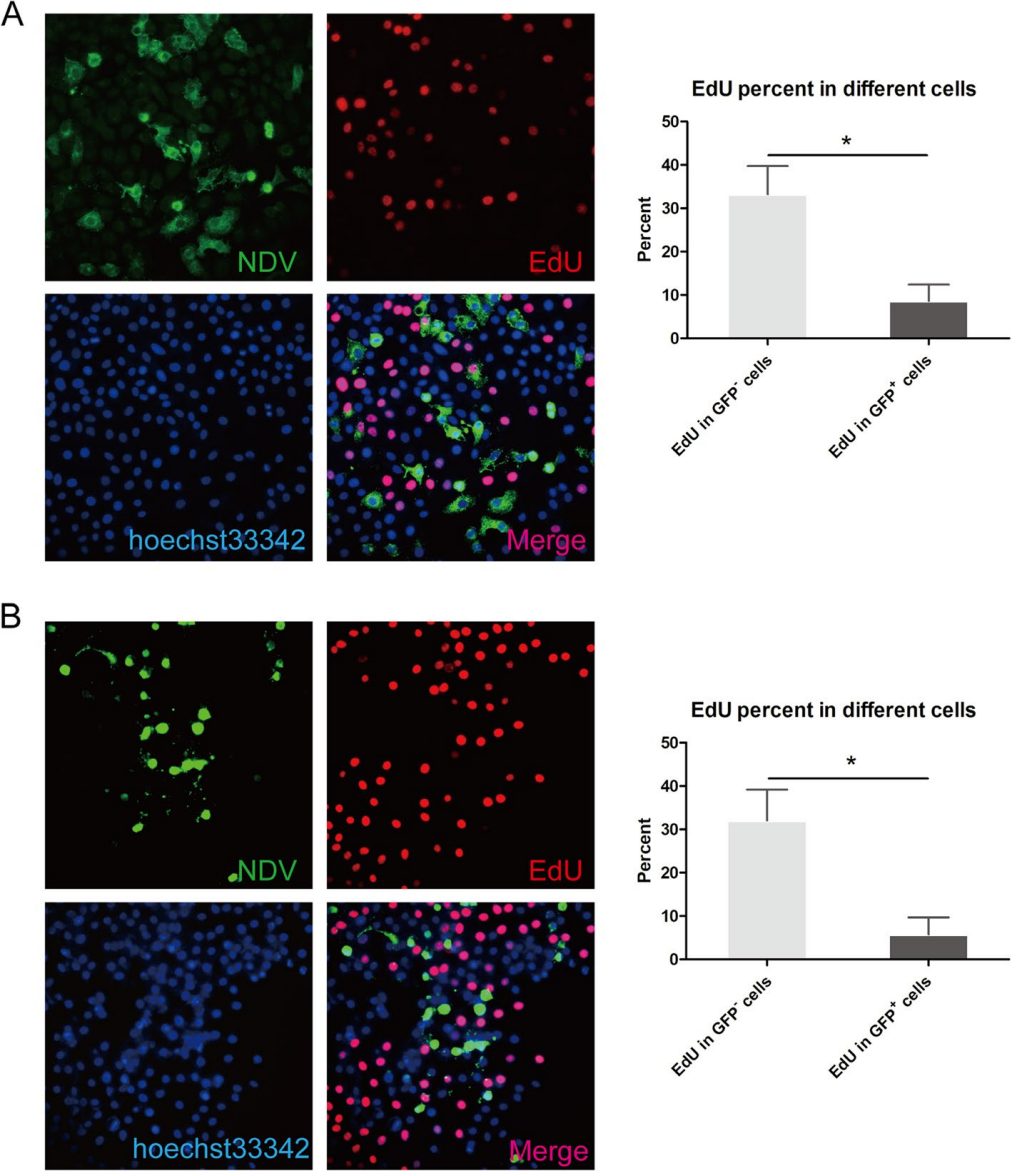
NDV inhibits cell proliferation at the single-cell level. HepG2 cells were infected with NDV. Then, 24 h (**A**) and 48 h (**B**) postinfection, the cells were labeled with EdU (10 nM) for 1 h and then stained with chicken anti-NDV serum and goat anti-chicken IgY Alexa Fluor® 488 (green) secondary antibodies. EdU staining was carried out according to the EdU manufacturer’s instructions. Nuclei were stained with Hoechst 33,342. Images were captured using a Leica fluorescence microscope (400 ×); bar = 50 μm

To determine the effect of NDV on cell proliferation at the single-cell level, we first infected HepG2 cells with NDV at a multiplicity of infection (MOI) of 1 for 24 h and then added 10 nM EdU to the cells and incubated these treated cells for 1 h. Then, the cells were subjected to immunofluorescence and EdU analyses, and the results showed that the proportion of EdU-positive and GFP-positive cells was significantly lower than the proportion of GFP-negative cells (Fig. 1A). Then, another dish of HepG2 cells was infected with NDV (MOI of 1) for 48 h, followed by immunofluorescence and EdU analyses (Fig. 1B). The single-cell level results were similar to those obtained after NDV infection for 24 h and suggested that NDV inhibited cell proliferation.

### The V protein promotes HepG2 cell proliferation

**Fig. 2:**
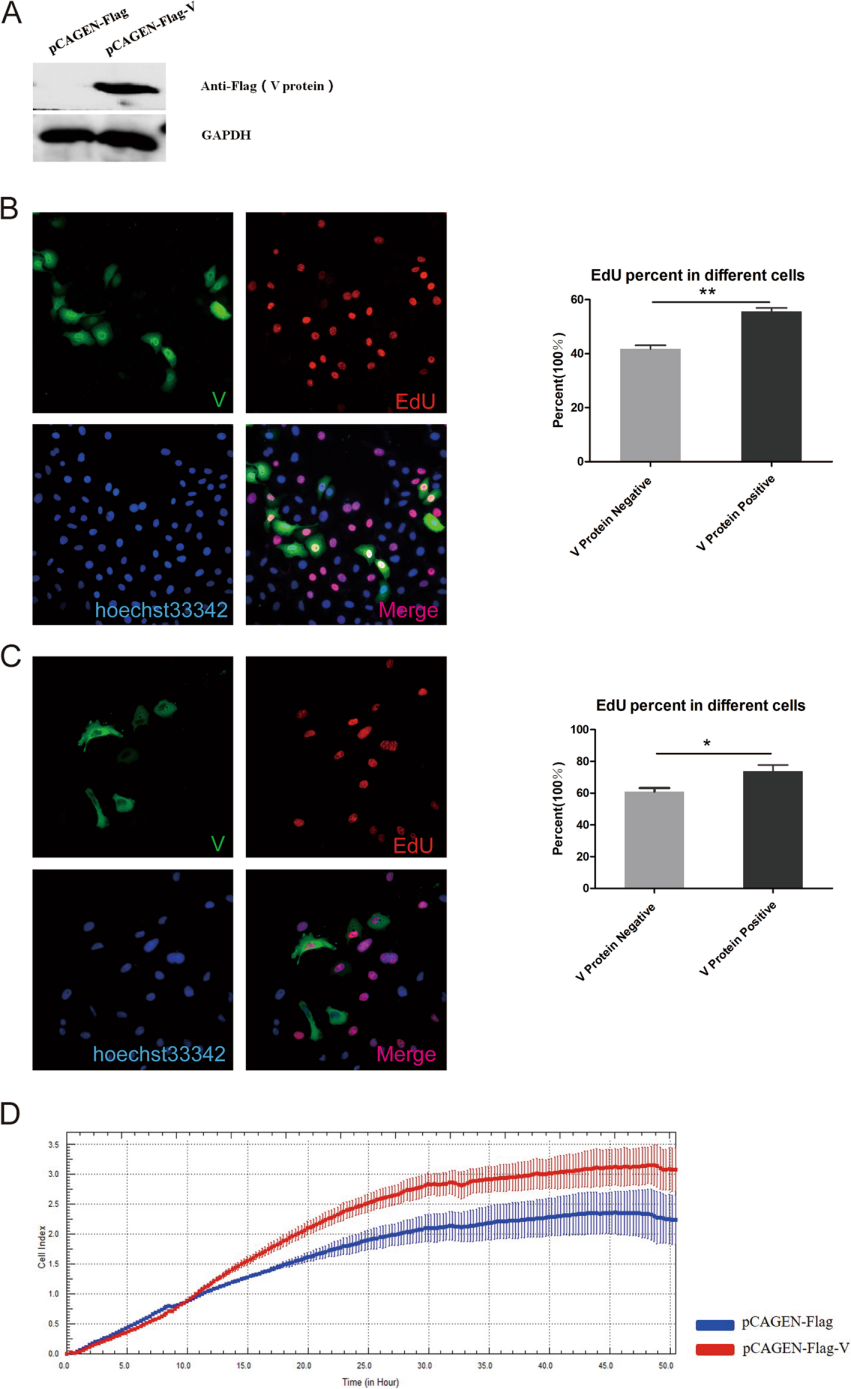
The V protein promotes cell proliferation at the single-cell level. HepG2 cells were transfected with pCAGEN-Flag-V (to overexpress the V protein). WB results showed that V protein was successfully overexpressed **A**. After 24 h (**B**) and 48 h (**C**), the cells were labeled with EdU (10 nM) for 1 h and then stained with rabbit anti-Flag antibody and goat anti-rabbit IgG Alexa Fluor.® 488 (green) secondary antibody. For EdU staining, the steps were carried out according to the EdU manufacturer’s instructions. Nuclei were subsequently stained with Hoechst 33,342. Images were captured with a Leica fluorescence microscope (400 ×); bar = 50 μm. The results of the unlabeled cell recorder showed that the number index of cells in the overexpression of V protein group was significantly higher than that in the control group (**E**)

To confirm the function of the V protein in cell proliferation, we overexpressed the V protein in HepG2 cells. Western blot (Fig. 2A and Supplementary Fig. 1) and immunofluorescence (Fig. 2B-C) analyses showed that the V protein was overexpressed in HepG2 cells. After transfection with pCAGEN-Flag-V for 24 h (Fig. 2B) or 48 h (Fig. 2C), 10 nM EdU was added and incubated for 1 h. The immunofluorescence and EdU analyses revealed more V protein-positive cells than V protein-negative cells. To verify this finding at the general level, we used a real-time label-free cell recorder to detect cell proliferation, and the results showed that the V protein promoted cell proliferation (Fig. 2D). These results suggested that the V protein enhanced HepG2 proliferation. However, compared with detection at 48 h after overexpression of the V protein (* *P* < 0.05), the effect of the V protein on promoting cell proliferation was more significant at 24 h (* * *P* < 0.01).

### Gene expression changes induced by the V protein

**Fig. 3:**
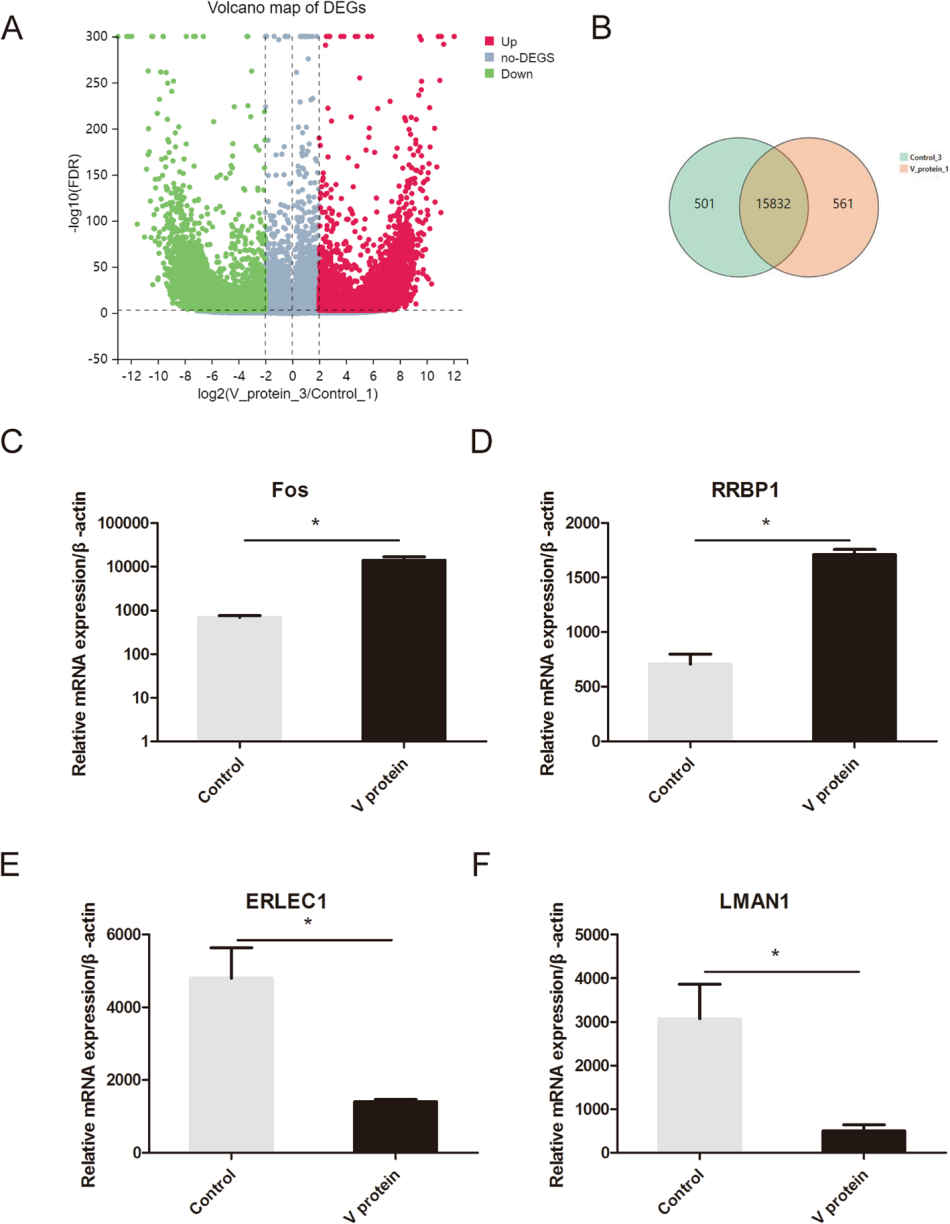
Differentially expressed genes (DEGs) in HepG2 cells overexpressing the V protein. RNA sequencing (RNA-seq) analyses of three independent experiments were repeated. **A** Volcano map showing differences in genetic transcription in HepG2 cells 48 h after transfection with pCAGEN-Flag-V. **B** Venn diagram analysis was performed to identify the genes with expression regulated through V protein overexpression. (**C**-**H**) qPCR detection of changes in gene expression after V protein overexpression. *P < 0.05

To determine the gene expression difference between the V protein overexpression group and the control group, sequencing was performed, and the results showed that the expression of genes was increased/downregulated (Fig. 3A). The green circle indicates the control group, and the red circle indicates the V protein overexpression group. A total of 561 genes were specifically expressed in V protein-overexpressing cells, and 501 genes were specifically expressed in control cells (Fig. 3B). qRT–PCR with β-actin as the reference revealed that the expression of the cell proliferation-related genes FOS and RRBP1 was increased and that the expression of ERLEC1 and LMAN1 was decreased (Fig. 3C-F); these results were consistent with the sequencing results.

### V protein through a variety of different signaling pathways to regulate cell proliferation

**Fig. 4:**
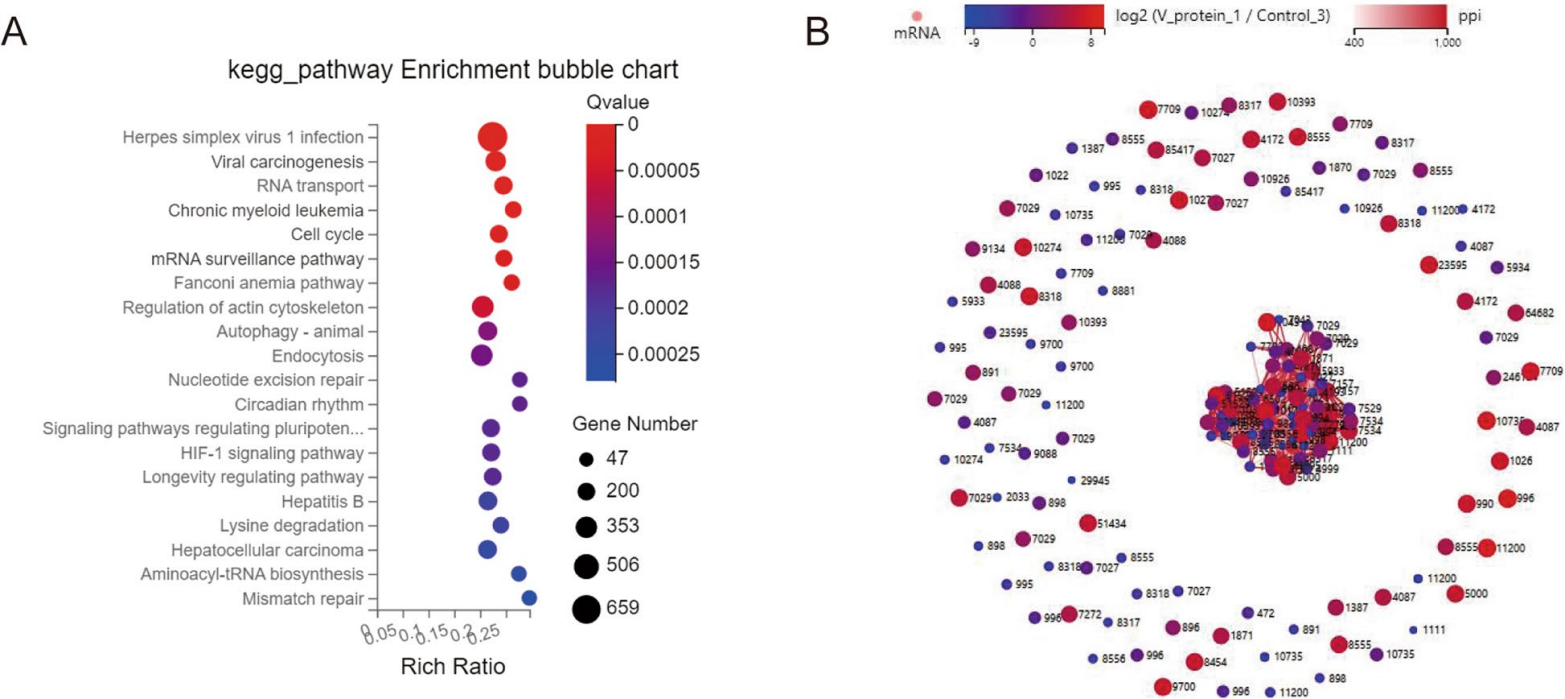
Gene Ontology (GO) terms and Kyoto Encyclopedia of Genes and Genomes (KEGG) pathways showing enriched differentially expressed genes (DEGs). **A** The KEGG pathways with the most significantly enriched DEGs. **B**, RNA sequencing (RNA-seq) results showed the relationship and expression changes of 166 cell cycle-related genes after overexpression of the V protein in HepG2 cells

An ingenuity pathway analysis showed significant changes in signaling pathways following V protein expression, and the 20 most significantly changed pathways are shown (Fig. 4A). NDV is a single-stranded negative-stranded RNA virus. Among the enriched pathways, herpes simplex virus 1 infection, viral carcinogenesis and RNA transport are closely related to viral cycle life, and changes in these signaling pathways suggested that the V protein plays a regulatory role in the viral life cycle. However, prior to this study, whether the V protein regulates cell proliferation, which affects changes in the cell cycle, was unknown. This study suggests a strategy for exploring the mechanism by which the V protein regulates cell proliferation. A total of 166 cell cycle-related genes were in the cell cycle signaling pathway (Fig. 4B). After V protein overexpression, the expression of most of these genes was increased, and they are marked in red (Fig. 4B). These results further demonstrate that the V protein can regulate cell proliferation.

### The V protein can regulate cell proliferation through multiple pathways

**Fig. 5:**
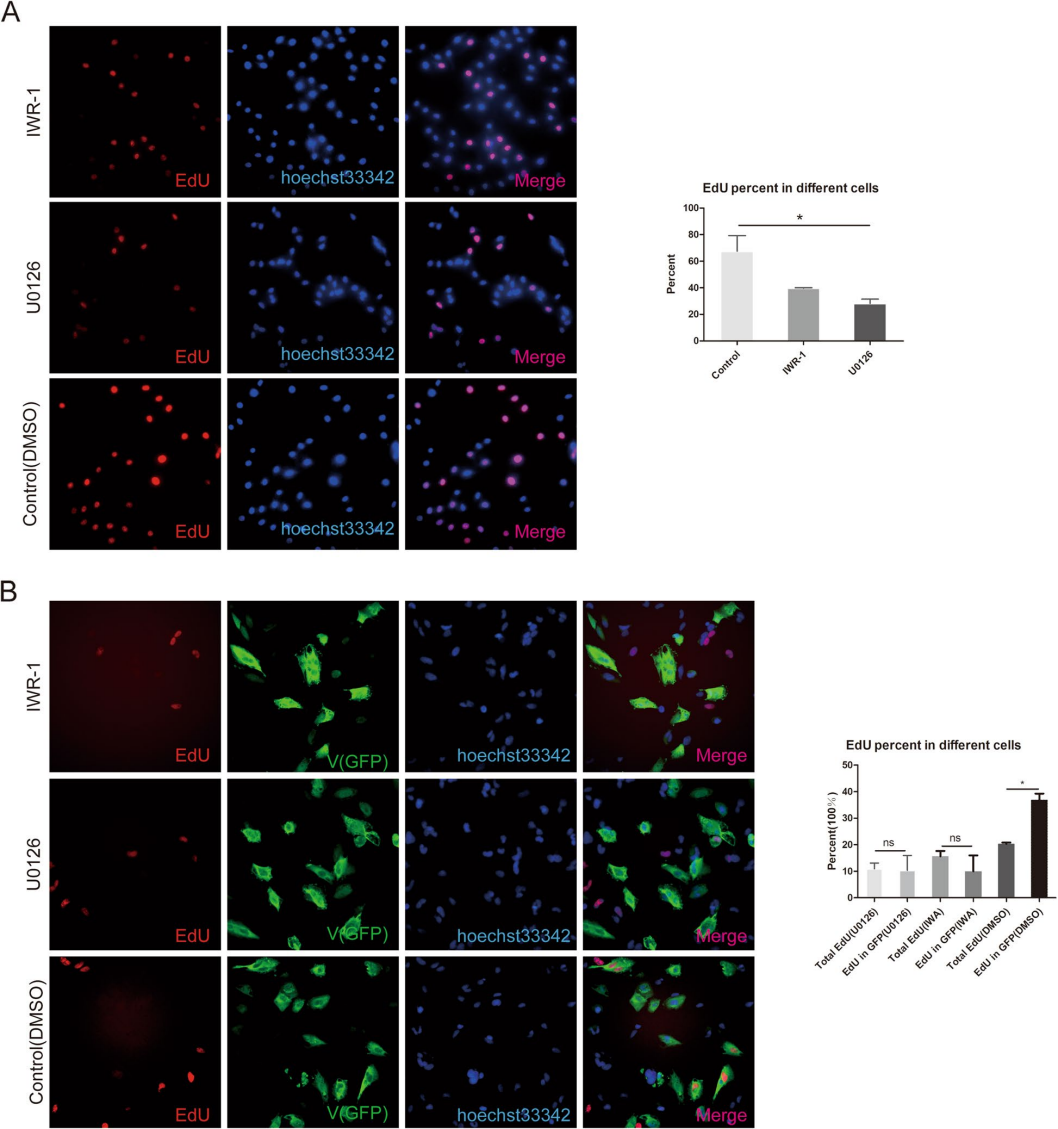
The MAPK and ERK1/2 signaling pathway inhibitor U0126 and WNT/β-catenin signaling pathway inhibitor IWR-1 interfere with the effect of V protein on cell proliferation. **A** After 6 h, U0126- and IWR-1-treated cells were labeled with EdU (10 nM) and incubated for 1 h. The EdU staining steps were carried out according to the EdU manufacturer’s instruction manual. Nuclei were subsequently stained with Hoechst 33,342, and EdU-positive cell percentages are shown on the right. HepG2 cells were transfected with pCAGEN-Flag-V (to overexpress the V protein). After 24 h (**A**), U0126 (**B**) or IWR-1 (**C**) was added to cells, which were incubated for another 6 h, followed by labeling with EdU (10 nM) and incubation for 1 h. Then, the cells were stained with rabbit anti-Flag antibody and goat anti-rabbit IgG Alexa Fluor® 488 (green) secondary antibody. For EdU staining, the steps were carried out according to the EdU manufacturer’s instructions. Nuclei were subsequently stained with Hoechst 33,342. Images were captured using a Leica fluorescence microscope (400 ×), bar = 50 μm

Many signaling pathways regulate cell proliferation. Although RNA-seq showed that the cell cycle was affected, no specific signaling pathways associated with cell proliferation regulation were enriched with the identified DEGs. Therefore, the mechanism through which the V protein regulates cell proliferation remains unclear. The MAPK and WNT signaling pathways are classical signaling pathways that affect cell proliferation, and variable genes caused by overexpression of V protein are partially concentrated in MAPK and WNT signaling pathways (Supplementary table). To verify that these signaling pathways affected the proliferation of cells after the V protein was overexpressed, we first used the ERK1/2 inhibitors U0126 (100 nM) and IWR-1 (200 nM) to examine the role played by the ERK1/2 and WNT pathways in HepG2 proliferation. The EdU results showed a reduction in the number of EdU-positive cells (red fluorescence) after treatment with 100 nM U0126 or 200 nM IWR-1 for 24 h compared with the control group (Fig. 5A).

Then, to verify that the changes in these two signaling pathways affected cell proliferation because of V protein expression, plasmids (pCAGEN-Flag-V) overexpressing the V protein were transfected into HepG2 cells, and inhibitors were added 24 h after transfection. For the cells treated for 12 h, EdU and immunofluorescence staining analysis showed that both U0126 and IWR-1 (Fig. 5 B) inhibited cell proliferation, as determined at the single-cell level. These results indicated that both signaling pathways affected cell proliferation through the action of the V protein. Hence, it is suggested that the V protein regulates cell proliferation through multiple mechanisms.

## Discussion

This study demonstrated at the single-cell level that NDV inhibited the proliferation of cancer cells; however, the V protein of NDV promoted the proliferation of cancer cells. These findings suggested that oncolytic NDV viral proteins may be involved in reducing the oncolytic effect, which provides a new perspective on oncolytic viruses.

NDV infects poultry, which can affect bursa development and can proliferate in susceptible chicken lines [14]. Dendritic cells bridge innate and adaptive immunity and regulate host resistance to viral invasion. When NDV infects mice, dendritic cells inhibit the proliferation of T cells [15]. In TC-1 cervical cancer cells, NDV reduced the apoptosis rate and suppressed cell growth [16].

Abdullah et al. [17] wrote a review on the abnormal proliferation and aggressive invasion behavior of GBM, which had been reported to be associated with aberrant Rac1 protein signaling. Notably, NDV interacts with Rac1 upon viral entry, syncytium induction, and actin reorganization in an infected cell during the replication process.

NDV potentially led to reactive oxygen species production in a dose-dependent manner, which might have led to NDV action against cervical cancer cell proliferation [18]. As an oncolytic virus, NDV affects cell proliferation, which is of interest. Our previous preliminary work showed, at the single-cell level, that NDV selectively infected dividing cells, which promoted NDV replication [19].

Our present study performed at the single-cell level showed that NDV infection resulted in cell proliferation inhibition, which may be detrimental to viral replication. However, we also found that at different times (24 h and 48 h) after NDV infection, there were proportional differences in the effect of the V protein on cell proliferation, suggesting that NDV may engage in other dynamic mechanisms to affect cell proliferation. Perhaps this difference gradually decreases with the extension of time, suggesting that V protein may promote cell proliferation at an early stage.

Vaccinia virus (VACV) has been reported to alter cell cycle regulation and trigger the host cell DNA damage response. Caroline K. Martin et al. concluded that VACV modulates host cell proliferation and host cell cycle progression through temporal expression of multiple VACV effector proteins [20]. The effects of the transactivator Tas encoded by foamy viruses and expressed after cell cycle arrest or before apoptosis highlight the different mechanisms through which virus–host interactions inhibit cell proliferation [21]. Encephalomyocarditis virus 2A protein inhibited apoptosis [22], and human papilloma virus 16 (HPV16) protein E7 increased PKM2 expression and activated the nonglycolytic function of PKM2 to promote cervical cancer cell proliferation [23]. However, no direct evidence has shown to date that the V protein encoded by NDV regulates host cell proliferation; however, our present work shows that the V protein promotes cancer cell proliferation.

Many mechanisms affect cell proliferation [24]. In this study, we demonstrated at the single-cell level that the V protein promoted cell proliferation. Transcriptome sequencing revealed the mechanism by which the V protein promotes cell proliferation. Namely, we found that the V protein activates/silences multiple genes in HepG2 cells and that these genes are enriched in multiple signaling pathways. This is the first study to perform RNA-seq to identify a signaling pathway through which the V protein regulates cell proliferation.

The MEK/ERK signaling pathway regulates cancer cell proliferation, apoptosis, inflammation, angiogenesis, metastasis and drug resistance. In our previous work, we found that the V protein promotes viral replication in HeLa cells through the activation of the MEK/ERK signaling pathways [10]. Moreover, NDV selectively infects dividing cells and promotes viral proliferation [19], and these previous studies suggest that viral proteins may regulate viral replication by affecting cell proliferation. In the present study, our results demonstrate that the V protein regulates cell proliferation through the ERK signaling pathway.

Similar to the MEK/ERK signaling pathway, the Wnt signaling pathway exhibits diverse functions. It is involved in an evolutionarily conserved cell-to-cell coordination mechanism and is crucial for a variety of physiological processes in an organism's body, including stem cell regeneration, proliferation, division, and migration; cell polarity and fate determination; and specification of neural crest, neural symmetry and morphogenesis [25]. For certain endogenous retroviruses, differential gene expression analysis of various TCGA datasets has revealed a link between HEMO expression and activation of the Wnt/β-catenin signaling pathway, particularly in endometrial cancer [26]. Wnt/beta-catenin signaling is activated in HPV ( +) cervicovaginal cells, and activation of the Wnt/beta-catenin signaling pathway may predispose organisms with early gene-encoded cellular factors to HPV infection [27]. However, the mechanism by which the V protein regulates cancer cell proliferation through the Wnt/beta-catenin signaling pathway remains unclear, although the present study has demonstrated that the V protein can regulate cell proliferation through the Wnt/beta-catenin signaling pathway.

Studies have shown that absence of the V protein may be an important measure to transform oncolytic viruses [28], and this finding does not contradict our findings, which indicate that the V protein promotes cell proliferation through multiple signaling pathways. In the future, more efficient oncolytic viruses may be obtained based on modification of the V protein.

## Conclusions

A well-understood phenomenon is the ability of oncolytic NDV to inhibit cancer cell proliferation. As a nonstructural protein of NDV, the V protein mainly plays a role in promoting virus replication, but our results show that the V protein can promote cancer cell proliferation at the single-cell level. This finding may suggest the idea of oncolytic virus transformation. Moreover, through transcriptome sequencing, we initially found some mechanisms by which the V protein promotes the proliferation of cancer cells, which may provide a new solution for better use of Newcastle disease virus for oncolysis. When killing cancer cells with viruses, it may be a good choice to delete the V protein from the viruses.

## Materials and methods

### Cell culture and virus

The HepG2 cells used in this experiment had been previously obtained and were stored in our laboratory (Shanghai EK-Bioscience Biotechnology Co., Ltd.). All cells were cultured in Dulbecco’s modified Eagle’s medium (DMEM; Thermo, Waltham, USA) supplemented with 10% fetal bovine serum (FBS; Gibco, Grand Island, USA) (2% FBS was used in the maintenance culture medium), 100 U/mL penicillin, 0.1 mg/mL streptomycin, 2 mM/L-glutamine (Invitrogen, Carlsbad, CA, USA), and 1% nonessential amino acids (Invitrogen, Carlsbad, CA, USA).

La Sota is an attenuated NDV strain that had been previously obtained and was stored in our laboratory. Viruses were propagated in the allantoic cavities of 9–11-day-old embryo-specific-pathogen-free chicken eggs, and the allantoic fluid was harvested and stored at − 70 °C until further use [10].

### Vector construction

The full-length V gene was amplified from NDV-infected cells and inserted into pCAGEN-Flag to generate pCAGEN-Flag-V. The sequences of the primers used are listed in sequence Table 1.

**Table 1:** Primers sequence

Gene Primers	Forward primer (5’to 3’)	Reverse primer (5’to 3’)
**FOS**	GGGGCAAGGTGGAACAGTTA	AGTTGGTCTGTCTCCGCTTG
**HSPA6**	GAGTGGCTGCCAAAAACTCG	GAGTGGCTGCCAAAAACTCG
**RRBP1**	CGCAAGGAGATGGCGAAAAC	GAGGACAGTCACATTGGGGG
**LMAN1**	GATCCAGGCAAAGGGGTCTC	TGTACTCGAAACGGCGATGT
**ERLEC1**	CCAGACAGTCAGGTGCAAAGA	CACTGGAGATTCAACCCCAAGA
**HSP90B1**	GTACGGATGGTCTGGCAACA	GTCTCTGATCAGCGGGTGTC
**β-actin**	GGGCATGGGTCAGAAGGATT	TCGATGGGGTACTTCAGGGT

### Transfection and viral infection

Kemix-TRLIP was used for plasmid transfection of HepG2 cells. After reaching a density of 60%, the cells were transfected. To overexpress the V protein, cells were transfected with pCAGEN-Flag-V, and the control cells were transfected with an empty pCAGEN-Flag plasmid. To examine the influence of V on cell proliferation, 24–48 h after transfection of the cells with the plasmid encoding the V protein, samples were collected for further analysis. At the appropriate time, whole RNA was collected and stored until further analysis.

### Flow cytometry

From 24 to 48 h posttransfection with pCAGEN-Flag-V, the number of apoptotic cells was detected by propidium iodide (PI) staining assay according to the manufacturer’s protocol. Briefly, 1 × 10^6^ cells were harvested and washed twice with PBS, fixed with precooled 75% alcohol for 2 h, washed twice with PBS, and incubated with 10 µl of PI in the dark for 30 min. The cells were analyzed with a FACSCalibur instrument (CytoFLEX, Beckman Coulter).

### Immunofluorescence analyses and EdU analysis

Cells were transfected with pCAGEN-Flag-V. Forty-eight hours later, 10 Nm EdU was added to the cultures and incubated for 1 h. The assay steps were performed according to the respective manufacturer’s instructions. Briefly, the cells were treated with paraformaldehyde solution, 0.3% Triton X-100, primary antibody (anti-Flag, CST, # 14793S, 1:200 for immunofluorescence; anti-NDV (chicken immune serum) polyclonal antibody, prepared in our laboratory, 1:1000 for immunofluorescence), Click additive solution, secondary antibody (goat anti-rabbit IgG H&L (Alexa Fluor® 488), Abcam, ab150077, 1:300 for immunofluorescence; goat anti-chicken IgY H&L (Alexa Fluor® 488), Abcam, ab150169, 1:300 for immunofluorescence) and Hoechst 33,342. Between every step, the cells were washed with PBS three times.

### RNA-seq and qRT–PCR

After overexpressing the V protein in HepG2 cells, total RNA was isolated with RNAiso Plus (Takara, Code No.: 9109). Sequencing libraries were generated using the NEBNext® UltraTM RNA Library Prep Kit for Illumina® (NEB, USA) following the manufacturer’s recommendations, and index codes were added to attribute sequences to each sample. The samples were sequenced with an Illumina HiSeq 4000 system (Illumina Inc., USA). Differential expression of transcripts in the treatment and control groups was measured. RNA-Seq was performed by BGI Co., and differentially expressed genes were identified with the linear models for microarray data (limma) package in GEO2R; the cutoff criteria were *P* < 0.05.

After overexpressing the V protein in HepG2 cells, the relative mRNA expression of C-Myc, ATF1, c-Fos, CCND1, NDV vRNA and C1QC was measured by qRT–PCR. The cells were lysed with TRIzol reagent (TaKaRa, Dalian, China) to obtain total cellular RNA. Subsequently, cDNA was synthesized by reverse transcription using the Prime Script RT reagent kit (TaKaRa, Dalian, China). qRT–PCR was performed with RealStar Green Fast Mixture (GenStar, Beijing, China) according to the manufacturer’s protocol. β-Actin was the internal control, and the relative expression of each gene was normalized to that of β-actin. Relative transcript levels were analyzed using the 2-△△T method. The sequences of the qRT–PCR primers are listed in Table 1.

### Real-time label-free cell recorder detection

A total of 10,000 cells per well were seeded on 16-well E-pates, and cell growth was recorded every 15 min for 96 h. Two hours after seeding the cells, a mixture consisting of a protein V expression vector and transfection reagent was added to the cells. Then, 0.2 µg of plasmid and 0.5 µl of transfection reagent were added to each well. After continuous recording, the experiments were replicated, and the results were analyzed.

### Statistical analysis

Statistical analysis was performed with GraphPad Prism 5 software (GraphPad Software, Inc., CA, USA). All values are expressed as the means ± SDs of three independent experiments. Student's t test and one-way ANOVA were used to evaluate the significance of differences; *P* < 0.05 was considered to be statistically significant.

## Supplementary Information

**Additional file 1.**

**Additional file 2.**

## Data Availability

The datasets used and/or analyzed during the current study are available from the corresponding author on reasonable request. The raw data from both the mRNA and miRNA sequencing were submitted to the GEO of NCBI with accession number PRJNA871355 for the mRNA datahttps://www.ncbi.nlm.nih.gov/sra/PRJNA871355).
