# Supplementary figures and images for: The V protein in oncolytic Newcastle disease virus promotes HepG2 hepatoma cell proliferation at the single-cell level
Source: BMC Cancer. 2023 Apr 17;23:346. doi: 10.1186/s12885-023-10815-4 (PMC10108501; doi:10.1186/s12885-023-10815-4)

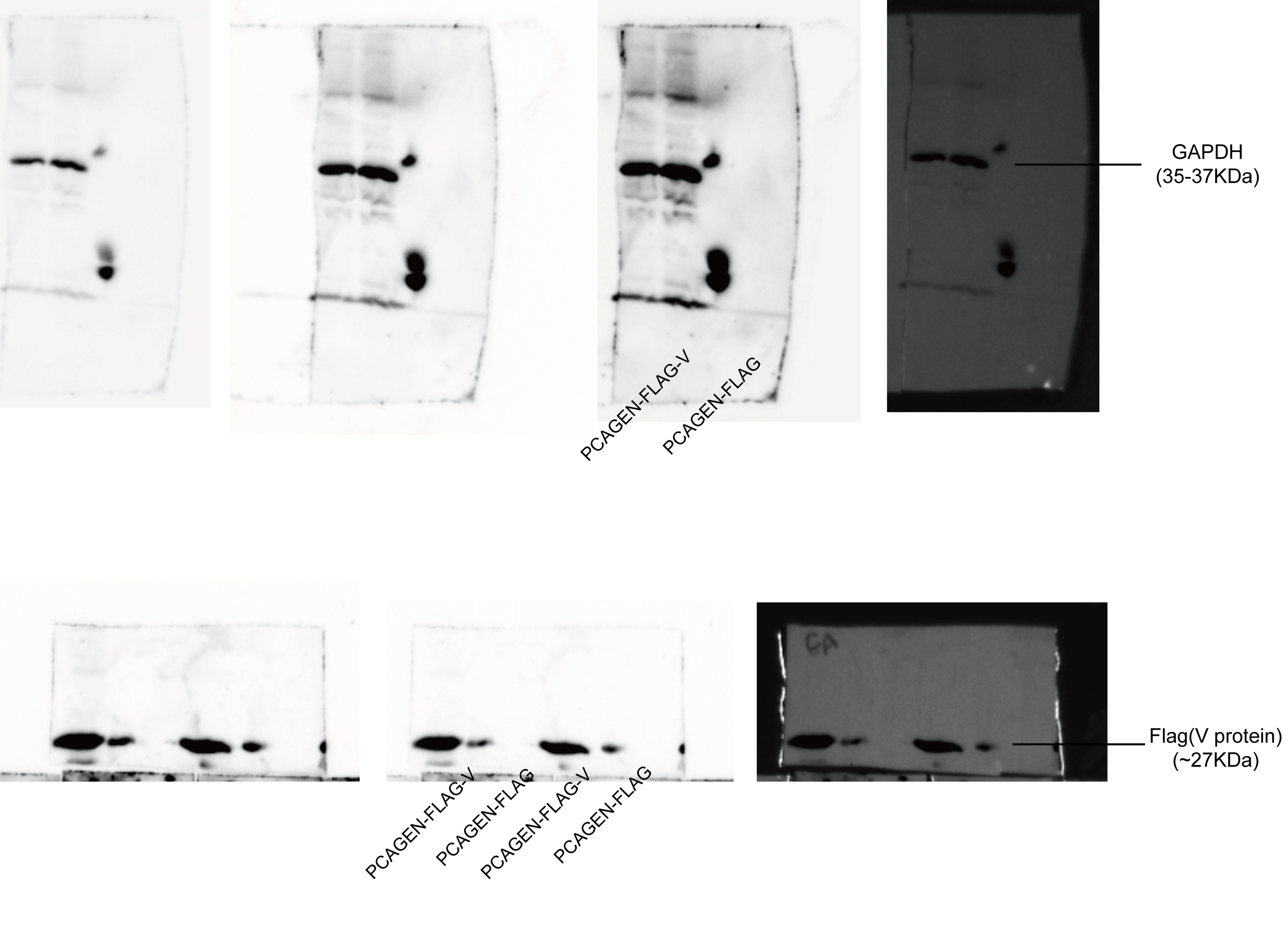

Supplement: Supplementary file 1 — Additional file 1. [file 12885_2023_10815_MOESM1_ESM.tif]
